# Negacionismo e positivismo

**DOI:** 10.1590/0102-311XPT144024

**Published:** 2024-11-04

**Authors:** Martinho Braga Batista e Silva

**Affiliations:** 1 Instituto de Medicina Social, Universidade do Estado do Rio de Janeiro, Rio de Janeiro, Brasil.

O dicionário ^1^ organizado pelos dois Josés - Szwako & Ratton - permite ao leitor diferenciar muitos fenômenos que podem se confundir, como negacionismo, fundamentalismo e conspiracionismo, ou mesmo informação incorreta, desinformação e má informação. Os mais de 100 verbetes que compõem o *Dicionário dos Negacionismos no Brasil* costumam primar pelas definições precisas, como é o caso do reacionarismo, distinto do conservadorismo. Essas distinções têm consequências, por exemplo, a caracterização do bolsonarismo como mais próximo do pensamento conservador, promotor de *fake news* e difusor de teorias da conspiração. Além disso, os autores apresentam tipologias cuidadosamente construídas, como é o caso dos três principais traços de anti-intelectualismo - irracionalismo, instrumentalismo e populismo - e os próprios seis tipos de negacionismo - científico, climático, dependente, histórico, estatístico e estrutural. Lendo alguns verbetes, aprendi como esse instrumentalismo marcou a Ditadura Militar no Brasil e atravessou o governo de Jair Bolsonaro, ambos avessos ao potencial explicativo e crítico das Ciências Humanas, ambos apegados ao “aplicado” por oposição ao “abstrato”. Além disso, percebi os traços de negacionismo histórico que marcaram os pronunciamentos desse governo sobre o período citado. A leitura desse dicionário certamente suscitará outros aprendizados e percepções entre integrantes da área de Saúde Coletiva. Não sendo possível sintetizar as quase 400 páginas que compõem a obra em uma resenha, optei por sinalizar aquilo que considero fundamental para a comunicação em saúde.

Paulo Freire, Bruno Latour, Giorgio Agamben, Naomi Oreskes e outros(as) intelectuais imprescindíveis para compreender um tema tão complexo merecem verbetes exclusivos, de maneira que o rigor de seus estudos sobre saber, ciência, Estado e mercado são expostos lado a lado de suas limitações e inclusive tropeços em investigá-los, como é o caso do teor conspiracionista identificado na reflexão sobre a pandemia de COVID-19 por parte de um dos mais citados filósofos contemporâneos. Isso sem falar dos(as) inúmeros(as) autores(as) citados(as) ao longo do desenvolvimento de argumentos no interior dos verbetes, como é o caso de Guy Debord, cuja obra sobre a espetacularização da vida comporta também contribuições específicas para compreender um dos elementos principais do fenômeno negacionista: a desinformação, ligada ao “*surgimento da mentira sem contestação*” (p. 107).

Instituições e não apenas indivíduos ocupam verbetes específicos, como é o caso do Instituto Brasileiro de Geografia e Estatística (IBGE) e outras conhecidas pela siglas - Fundação Oswaldo Cruz (FIOCRUZ) e Sociedade Brasileira para o Progresso da Ciência (SBPC) - e a Associação Brasileira de Ciências e a Rede Brasileira de Mulheres Cientistas. Inclusive, a maior parte dos(as) autores(as) dos verbetes se concentram em algumas instituições universitárias - quase 20 na Universidade Federal de Pernambuco (UFPE), mais de dez na Universidade do Estado do Rio de Janeiro (UERJ) - totalizando mais de 30 instituições de Ensino Superior nacionais e sete internacionais, sem falar nos institutos, nas fundações e outras entidades às quais eles(as) pertencem.

Os complementos entre parênteses ao lado de alguns verbetes também chamaram a minha atenção: anti, ataque aos, combate a, gestão da, formas pelas quais temas como constitucionalismo, ativismo, desinformação e a própria pandemia no Brasil foram apresentados, destacando a postura do Governo Federal no período de 2018-2022, belicosa para dizer o mínimo. Inclusive, a pandemia demanda mais de um verbete, de maneira que suas múltiplas facetas - sanitária, humanitária, administrativa, histórica e social - ganhem visibilidade no interior do processo de entendimento acerca de um fenômeno majoritariamente visto como de ordem epidemiológica. É assim que além de dois verbetes explícitos sobre o tema - pandemia de COVID-19 e pandemia no Brasil (gestão da) - nota-se a relevância de outros, sobre sindemia, por exemplo.

Participação, essa categoria ao mesmo tempo teórica e prática, um procedimento e um valor, foi tipologizada pelo autor e pela autora de um dos verbetes por meio de experiências nacionais de participação social, distribuindo-as em seis grupos: os orçamentos participativos, as instâncias de planejamento urbano, as instâncias de gestão de recursos hídricos, as audiências públicas, os conselhos comunitários e, finalmente, as conferências nacionais de políticas públicas. Relevante na área da saúde, a participação da comunidade é um dos princípios constitucionais do Sistema Único de Saúde (SUS) e aponta justamente para a possibilidade de fiscalizar a oferta de serviços de saúde nos conselhos e de elaborar propostas que se tornem políticas públicas nas conferências. Segundo o verbete sobre participação social, as conferências “*podem ser mandatórias por legislação setorial de nível federal ou convocadas discricionariamente pelo executivo federal para explorar possibilidade de se gerar acordos sobre mudanças na política em uma determinada comunidade de políticas*” (p. 242). Qual teria sido o caso da 17ª Conferência Nacional de Saúde, em julho de 2023? E da 5ª Conferência Nacional de Saúde Mental, em dezembro do mesmo ano? Qual será o da 4ª Conferência Nacional de Gestão do Trabalho e da Educação em Saúde?

Em alguns trechos do *Dicionário dos Negacionismos no Brasil*, percebo que o esforço de apresentar as características do negacionismo termina por disseminar uma perspectiva positivista da ciência. Essa tendência não é exclusiva desse dicionário, já que esse fenômeno foi comum durante a pandemia de COVID-19: “*Temos vivido polarizações entre o negacionismo e o obscurantismo, de um lado, e a reificação da ciência, de outro. Polarizações são sinais de ausência de mediações para compreendermos o fenômeno. Se a primeira postura, anticientífica, tem caracterizado o atual governo e seus apoiadores, a segunda tende ao neopositivismo e à visão de neutralidade científica*” ^2^ (p. 155-6).

Opor estatísticas a falas governamentais, apresentar dados científicos quantitativos como caminho privilegiado para argumentar contra opiniões, acusar de populismo um conjunto de tentativas para evitar a hierarquização de conhecimentos naturalísticos, tradicionais, populares e científicos, entre outros momentos nos quais há um certo neopositivismo permeando os densos debates, evidentemente são exceções, embora não deixem de vigorar em alguns verbetes. Trata-se de algo possível em uma obra abrangente, uma tendência que cabe em uma coletânea polifônica. Ainda assim, fica o alerta: “*O que nós, cientistas ou não, não podemos fazer, é atualizar a falsa ideia, tanto positivista como iluminista, de que há algo como uma evolução do pensamento humano, de uma fase mais simples e tosca, para uma fase mais elaborada e sofisticada, de modo que as outras formas de pensamento serão dirimidas, assimiladas ou simplesmente, desaparecerão, como chegou-se a afirmar em séculos passados do suposto duelo entre ciência e religião*” ^3^.

O dicionário suscita muitos aprendizados e nada disso que foi mencionado no parágrafo anterior diminui o valor inestimável que ele tem para o SUS, as Ciências Humanas e todos(as) os(as) envolvidos(as) na transformação da realidade na contemporaneidade. A obra foi semifinalista do 65^o^ Prêmio Jabuti em 2023 e a importância desses verbetes para a Saúde Coletiva é vasta, já que abordam assuntos próprios da área de conhecimento, como a pandemia de COVID-19, e aqueles que permeiam suas subáreas, como participação social em saúde, também explicando conceitos centrais para compreender o processo saúde-doença na atualidade, como é o caso das *fake news*. O ineditismo do *Dicionário dos Negacionismos no Brasil* é inegável, os organizadores e autores(as) prestaram um serviço incrível para o ensino de várias disciplinas e o esclarecimento de vários públicos, inclusive inovando no que diz respeito à comunicação pública da ciência.
